# Early Appearance of TNF-α and Other Cytokines in Bronchus Associated Lymphoid Tissues (BALT) from Growing Wistar Rats. What is the Role of TNF-α?

**DOI:** 10.1080/17402520400001686

**Published:** 2004

**Authors:** Dreanina Delettieres, Cecilia A. Frecha, Maria E. Roux

**Affiliations:** Faculty of Pharmacy and Biochemistry Laboratory of Cellular Immunology Department of Biological Sciences University of Buenos Aires, Junin 956, 5th floor Buenos Aires C1113AAD Argentina

## Abstract

Several different cytokines trigger the development of determined cell subsets in BALT of growing Wistar rats. Early appearance (4 days post partum) of γδT cells in BALT has been shown, as well as its role in up-regulating TNF-α production. In the present report, we studied in the BALT: (1) the profile of the cytokines, TNF-α, INF-γ and IL-10 and (2) in TCR γδ+ cells, the existence of a colocalization with TNF-α as well as with INF-γ. All the cytokines studied were observed at an early stage of BALT development by immunohistochemistry and in bronchoalveolar cells (BAL cells) by flow cytometry and western blot. (1) The principal cytokine found at 4 days of age in BALT cells was TNF-α that increases along BALT development. The same behavior was found for cells containing IL-10 and INF-γ. (2) TCR γδ+ cells colocalize mainly with TNF-α as it has been shown by immunohistochemistry in BALT and by flow cytometry when we studied BAL.

The early appearance of TNF-α concomitant with TCR γδ+ cell suggests an important role for this cytokine along BALT development. Moreover, mutual regulation between them exists taking part in the immune surveillance and repair of damaged epithelia.


*Clinical & Developmental Immunology*, September/December 2004, VOL. 11 (3/4), pp. 253–259

Taylor & Francis healthsciences

### Early Appearance of TNF-α and Other Cytokines in Bronchus Associated Lymphoid Tissues (BALT) from Growing Wistar Rats. What is the Role of TNF-α?

DREANINA DELETTIERES, CECILIA A. FRECHA and MARIA E. ROUX*

*Faculty of Pharmacy and Biochemistry, Laboratory of Cellular Immunology, Department of Biological Sciences, University of Buenos Aires, Junin 956, 5th floor, C1113AAD Buenos Aires, Argentina*

Several different cytokines trigger the development of determined cell subsets in BALT of growing Wistar rats. Early appearance (4 days post partum) of γδT cells in BALT has been shown, as well as its role in up-regulating TNF-α production. In the present report, we studied in the BALT: (1) the profile of the cytokines, TNF-α, INF-γ and IL-10 and (2) in TCR γδ+ cells, the existence of a colocalization with TNF-α as well as with INF-γ. All the cytokines studied were observed at an early stage of BALT development by immunohistochemistry and in bronchoalveolar cells (BAL cells) by flow cytometry and western blot. (1) The principal cytokine found at 4 days of age in BALT cells was TNF-α that increases along BALT development. The same behavior was found for cells containing IL-10 and INF-γ. (2) TCR γδ+ cells colocalize mainly with TNF-α as it has been shown by immunohistochemistry in BALT and by flow cytometry when we studied BAL.

The early appearance of TNF-α concomitant with TCR γδ+ cell suggests an important role for this cytokine along BALT development. Moreover, mutual regulation between them exists taking part in the immune surveillance and repair of damaged epithelia.

*Keywords*: Development of BALT; Cytokines; Growing Wistar rats; BAL; TNF-α+ TCR γδ+ cells

### INTRODUCTION

The notion of the lung as an immunological organ was developed years ago and it has been described in several reviews (Sminia *et al.*, 1989; Bienenstock *et al.*, 1999). The organized lymphoid tissue in the lung is called bronchus associated lymphoid tissue (BALT) and is located along the main bronchi, near the bifurcation in all the lobes (Bienenstock *et al.*, 1999).

The sequential study of the appearance of the different T cell subsets, starting at day 4 postpartum and especially of γδ+ T cell has been done in our laboratory, since the first BALUs appear at that age (Marquez and Roux, 1998; Marquez *et al.*, 2000).

The fact that γδ T cells have been noted in many situations in which TNF-α is produced is consistent with the notion that this cytokine is triggering γδ T cell reactivity. Since γδ T cells themselves can produce TNF-α, they might also be auto stimulatory under appropriate conditions (Lahn *et al.*, 1998). Other studies have provided evidence for the role of γδ T cells in up-regulating TNF-α production, suggestive of a feed back loop in which γδ T cells could perpetuate the generation of their own major stimulus (Nishimura *et al.*, 1995).

Due to the fact that there are no previous works done on lung cytokines we thought it could be of interest to study the early development of TNF-α, IFN-γ and IL-10. Besides knowing how TNF-α behaves during BALT ontogeny we asked ourselves if there exists a colocalization between the above cytokine or INF-γ with the TCR γδ phenotype.

### MATERIALS AND METHODS

### Animals

Conventionally housed suckling 4, 7 and 12-days-old weaning rats and 60-days-old rats of the Wistar strain (closed colony from the breeding unit kept at the animal facilities of the Faculty of Pharmacy and Biochemistry, University of Buenos Aires, Argentina) of either sex were used. Weaning rats (21 days old) were fed a stock diet (Cargill Argentina, 24.6% protein) up to 60 days of age. Water and diet were given *ad libitum*. During all the experimental time

*Corresponding author. Address: Ugarteche 3050 1st Apartment, 42, C1425EVF Buenos Aires, Argentina. Tel.: +54-11-4801-2864. Fax: +54-11-4508-3645. E-mail: mroux@ffyb.uba.ar

ISSN 1740-2522 print/ISSN 1740-2530 online © 2004 Taylor & Francis Ltd
DOI: 10.1080/17402520400001686

animals were subjected to a 12 h light–darkness cycle, room temperature was kept at 21 ± 1°C.

Experiments were performed using 5 or 6 animals per group. This project has been approved by the University of Buenos Aires Ethical Committee.

### Tissue Sections

The lower respiratory tract was removed and placed in 95°C ethanol at 4°C in order to be processed by Sainte-Marie's technique (Sainte-Marie, 1962). Briefly, tissues were fixed in 95°C ethanol, pre-cooled at 4°C, dehydrated in four changes of pre-cooled absolute ethanol, cleared by passing through three consecutive baths of xylene and embedded in paraffin at 56°C. Tissue sections (4–5 μm thick by microtome) were placed on glass microscope slides.

### Immunohistochemistry

### *Indirect Immunofluorescence*

Paraffin from BALT tissues section on glass microscope slides was removed by gently swirling the slides in three consecutive baths of xylene which was removed in two baths of absolute ethanol, two baths of 95°C ethanol and two baths of saline solution (0.9% NaCl w/v in distilled water).

Cytokines and cell markers were studied on tissue sections of the lower respiratory tract of 4, 7, 12, 21 and 60-day-old rats by an indirect immunofluorescence.

Tissue sections of BALT were viewed at 1000× magnification with an Olympus epifluorescence microscope.

TNF-α + and IFN-γ + cells were detected by simple indirect immunofluorescence technique using (1) high affinity purified IgG rabbit anti rat TNF-α (Pepro Tech, NJ, USA) after an overnight incubation at 4°C the FITC-conjugated goat F $(ab)'_2$ fragment to rabbit IgG (Whole molecule) (ICN Cappel, Aurora, OH, USA) and (2) high affinity purified IgG goat anti- rat INF-γ (Pepro Tech INC, NJ, USA) after an overnight incubation at 4°C, the FITC-conjugated rabbit F (ab)′2 fragment to Goat IgG (Whole molecule) (ICN Cappel, Aurora, OH, USA). All tissue incubation was performed in a moist chamber, and sections were washed three times in saline after each incubation step. Control staining was carried out simultaneously omitting the primary antibody step.

TCR γδ+ /TNF-α+ and IFN-γ+ /TCR γδ+ populations were labeled performing a double fluorescence technique. The antibodies used were monoclonal mouse IgG to rat TCR γδ (Clone V65) (BD PharMingen, San Diego, CA, USA) for an overnight incubation and then after three washes, in saline solution. Alexa Fluor 488 Dye goat IgG anti-mouse IgG (highly cross-adsorbed) (Molecular Probes, USA) was added for half an hour. After three washes in saline solution, sections were incubated with high affinity purified IgG rabbit anti-rat TNF-α (Pepro Tech INC, NJ, USA) for 6 h and after three washes, with saline solution they were incubated with Alexa Fluor Dye 546 goat IgG anti-rabbit IgG (highly cross–adsorbed) (Molecular Probes, USA) for 1 h.

Then tissue sections were washed three times and mounted.

The same technique was followed to detect TCR γδ+ cells/INF-γ + cells using the appropriate antibodies.

Cell count was performed by the authors in a blind fashion.

### Statistical Analysis

Results are expressed as mean ± standard error (SE) of the absolute number of cells in BALT lamina propria.

Due to the very small size of BALT in 4-day-old as well as 7-day-old rats one field was recorded and for 12, 21 and 60-day-old rats15 fields were studied.

Statistical evaluation of results was with Graph Pad InStat version 3.01 (Graph Pad Software, San Diego, CA, USA) using two tailed Student's *t*-test, and one way ANOVA with Tukey–Kramer's post test, taking $p < 0.05$ as significant.

### Flow Cytometry Studies

### *Isolation of Cells from BAL*

At each time point, groups of four animals were studied. For bronchoalveolar (BAL) lavage the thorax was opened, the diaphragm pierced and the anterior rib cage removed. A blade was used to make a small transverse aperture between the cricoid cartilage and the first cartilage ring of the trachea. After careful haemostasis, 1–22 mm portex tubing (TUB 3736, Scientific Laboratory Supplies) was passed into the trachea to just above the carina.

BAL cells were immediately diluted in cold RPMI containing 10% fetal calf serum, 2 mm/ml L-glutamine, penicillin (50 U/ml) and streptomycin (50 ug/ml).

### *Cell Surface Antigen and Intracellular Cytokine Staining*

Freshly isolated cells were stimulated with 50 ng/ml PMA (Sigma) and 500 ng/ml ionomycin (Calbiochem) at 37°C. To avoid adherence, all cell incubations were performed in non-tissue culture grade plastic tubes. Brefeldin A (10 ug/ml) (Epicentre Technologies) was added for the last 2 h of incubation to disaggregate the Golgi complex and intracellular cytokines were allowed to accumulate. Once stimulated, cells were harvested and washed in ISOTON (from Coulter Burlington, Ontario).

For cell surface staining cells ($10^6$) were incubated with FITC-conjugated mouse $IgG_{1k}$ anti-rat TCR γδ (clone V65) (BD PharMingen, San Diego,CA, USA) for 30 min at 4°C in the dark. Cells were washed once with 2 ml of ISOTON and spun at 400*g*, 4°C for 5 min. They were fixed with 1% formaldehyde in PBS for 20 min at room temperature. They were stained immediately for cytokines as described by Openshaw *et al.*, (1995), Caraher *et al.* (2000) and Pala *et al.* (2000). Briefly, for intracellular staining, cells were permeabilized with 0.5% saponine (saponine buffer) for 10 min at room temperature. Cells were treated with the corresponding antibody (PE anti TNF -α or PE Anti-INF-γ or Isotype matched monoclonal antibody) (BD PharMingen, San Diego, CA, USA) for 30 min at 4°C in the dark.

Cells were washed with saponin buffer twice and once with ISOTON. Then they were spin down at 1500 rpm. Supernatant was discarded and the pellet was resuspended in 2% paraformaldehyde in PBS and stored in the dark at 4°C. Samples were run on a FACS flow cytometer (Becton Dickinson, San Jose, CA, USA). Data were collected for 10,000 events. Data analysis was performed using Lysys II software by Becton Dickinson and WinMDI 2.3 software.

### Statistical Analysis

Results are reported as mean ± SE. Statistical analyses were performed by two-tailed Student's *t*-test conducted using InStat (Graph Pad Software, San Diego, USA).

### Western Blot Analysis

Cytosol fractions were isolated from Broncheal Alveolar cells (Buckley *et al.*, 1988). The presence of TNF-α and IFN-γ in the cytosol fractions was detected using Western blot analysis.

Briefly, the cytosol fractions were run on Polyacrilamide gel containing SDS (Laemmli, 1970) using a wide molecular weight standard from 17–250 kd after they were transferred electroforetically to PVDF membrane (Hybond-P, Amersham Pharmacia Biotech) (Towbin *et al.*, 1979).

To avoid background as well as unspecific reactions the membrane was incubated with PBS buffer pH 7.4 containing sodium azide 0.02 and 10% albumin.

To confirm the presence of all the cytokines studied we used Avidine-Biotin-Peroxidase DAB (Vectastain® ABC Kit, Vector Laboratories, Inc, Burlingame, CA, USA). Therefore to detect TNF-α, INF-γ and IL-10, the following highly purified antibodies were used: rabbit IgG against rat TNF-α, goat IgG against rat INF-γ and rabbit IgG against rat IL-10 (Pepro Tech INC, NJ, USA).

After an overnight incubation at 4°C the membranes were washed three times and incubated for 1 h at room temperature with the second corresponding antibody, biotinylated goat anti rabbit IgG or biotinylated rabbit anti Goat IgG (Vector Laboratories, CA, USA). Finally the immune reaction band in each case was detected using Biotin Avidin Peroxidase complex. The ABC system was revealed with DAB (Sigma).

### RESULTS

### Immunohistochemistry: Indirect Immunofluorescence

Figure 1 shows TNF-α positive cells found in BALT at 4 days of age while Fig. 2 shows at 60 days of age. Figure 3 shows the colocalization between TNF-α and TCR γδ observed as early as 7 days of age. In contrast, fewer double positive IFN-γ/TCR γδ cells were observed.

Table I represents the number of cells containing the following cytokines: TNF-α, IFN-γ and IL-10 at 12, 21 and 60 days of age. The results are expressed as mean ± SE of the number of cells in 15 fields from 5 animals. (1) TNF-α: 15.53 ± 2.9 vs. 105.6 ± 10.8 vs. 155.3 ± 20.6. Tukey–Kramer test indicated significant

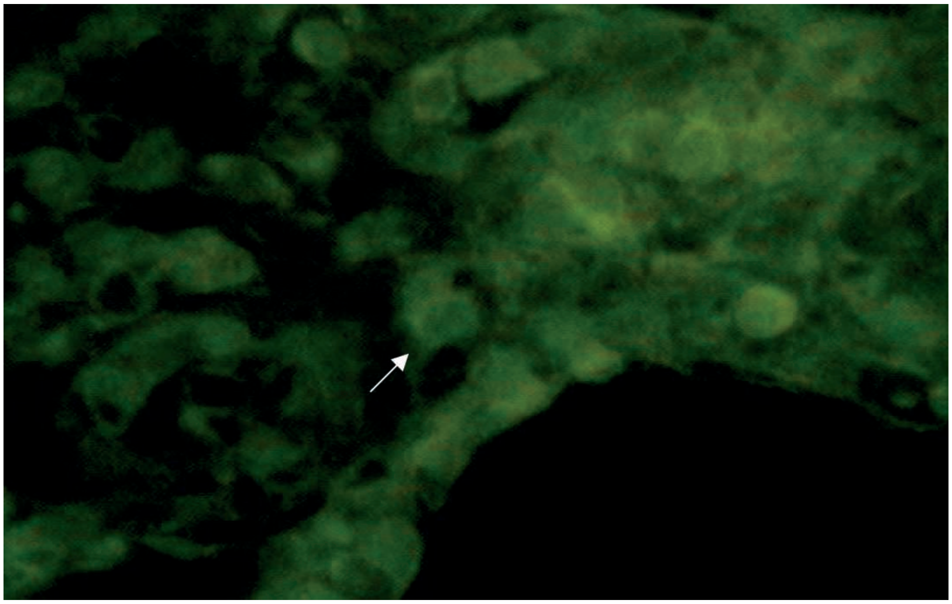

FIGURE 1 Immunofluorescence staining of paraffin section from BALT of 4-days-old rats with anti TNF-α magnification 1000 ×. Arrow indicates a TNF-α + cell.

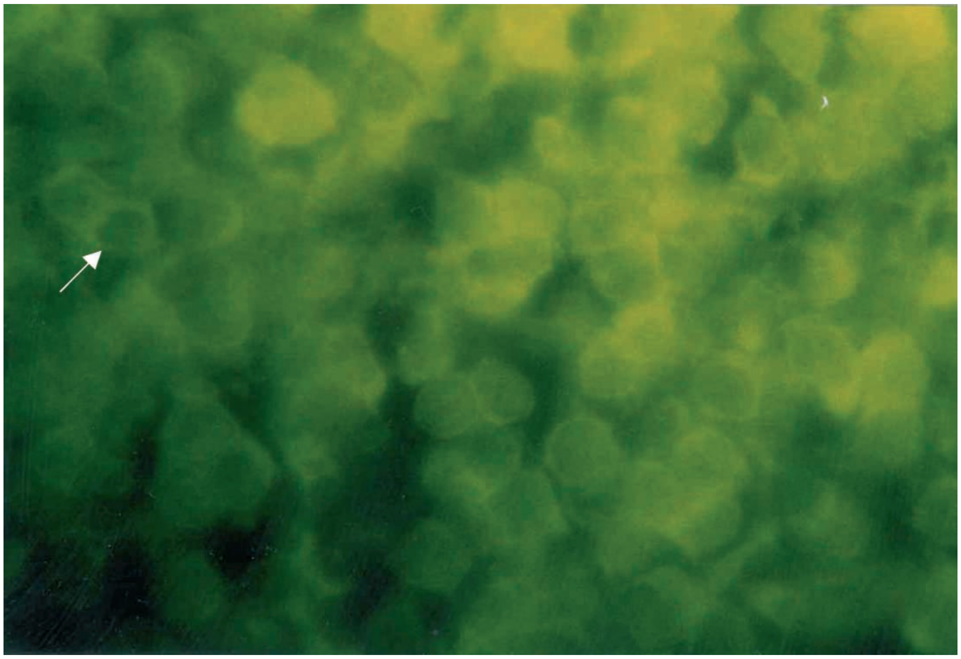

FIGURE 2 Immunofluorescence staining of paraffin section from BALT of 60-days-old rats with anti-TNF-α magnification 1000 ×. Arrow indicates a TNF-α + cell.

differences between 12 vs. 21 ($p < 0.01$) and 12 vs. 60 ($p < 0.001$). (2) INF-γ: 83.4 ± 5.1 vs. 217 ± 72 vs. 344.5 ± 49.5. Tukey–Kramer test indicated significant differences only between 12 vs. 60 ($p < 0.01$). (3) IL-10: 57.36 ± 13.6 vs. 120.6 ± 10.2 vs. 178.2 ± 14.5. Tukey–Kramer test indicated significant differences 12 vs. 21 ($p < 0.05$) 12 vs. 60 ($p < 0.01$) and 21 vs. 60 ($p < 0.05$). In this table, we observed that all the cytokines studied increase during BALT development.

Figure 4 and Table II describes that a high TNF-α/IL-10 ratio at day 4 reverses at day 21. In contrast, the IFN-γ/IL-10 ratio is always greater than one.

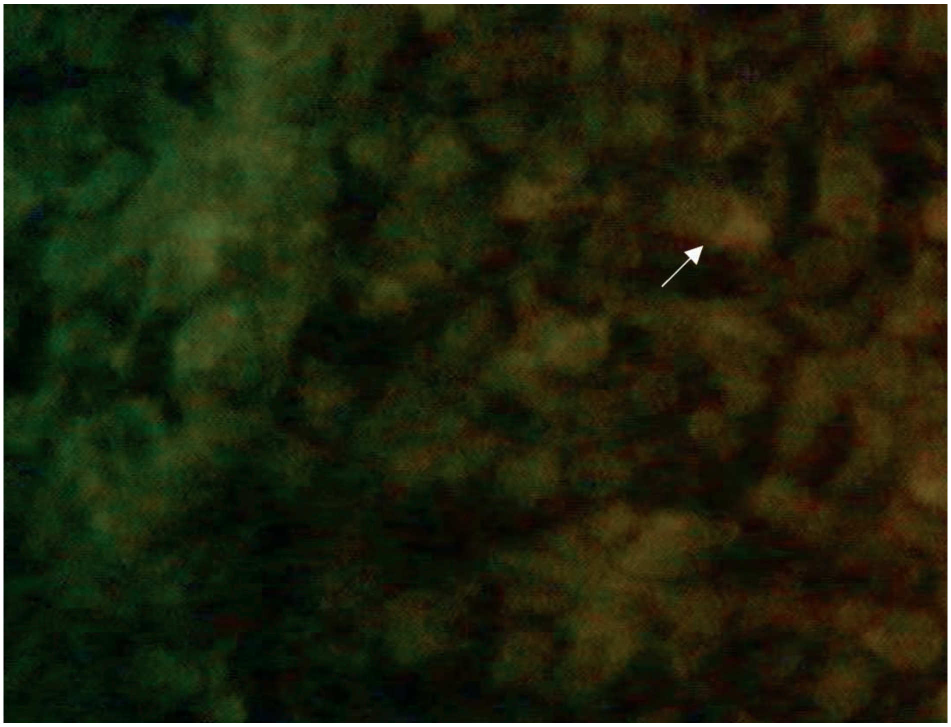

FIGURE 3 Immunoflurescence staining of paraffin section from BALT of 7-days-old rats showing TCR γδ + cells (green), TNF-α + cells (red) and TCR γδ + TNF-α + cells yellow magnification 1000 ×. Arrow indicates a TCR γδ + TNF-α + cells.

TABLE I Number of TNF-α+ , INF-γ+ and IL-10+ cells in rats BALT

| | Cytokines | | |
|---|---|---|---|
| Days of age | TNF-α | INF-γ | IL-10 |
| 12 | 15.53 ± 2.9* | 83.4 ± 5.1** | 57.36 ± 13.5*** |
| 21 | 105.6 ± 10.8 | 217 ± 72.0 | 120.6 ± 10.2# |
| 60 | 155.3 ± 20.3 | 244.5 ± 49.5 | 178.2 ± 14.5 |

Mean ± SE of the number of cells in 15 fields from 5 animals.
*12 vs. 21 $p < 0.01$, 12 vs. 60 $p < 0.001$; **12 vs. 60 $p < 0.01$; ***12 vs. 21 $p < 0.05$, 12 vs. 60 $p < 0.001$; # 21 vs. 60 $p < 0.05$.

### Flow Cytometry Analysis

Figure 5(A) indicates the percentage of PE-TNF-α + cells (1.56%), the percentage of PE-TNF-α + cells expressing TCR γδ phenotype (2.45%) and the percentage of FITC-TCR γδ phenotype (11.28%) at 4 days of age. Figure 5(B) indicates the percentage of PE-TNF-α + cells (1.62%), the percentage of PE-TNF-α + cells expressing TCR γδ phenotype (2%) and the percentage of FITC-TCR γδ phenotype (3.74%) at 60 days of age.

### Western Blot Analysis

TNF-α, INF-γ as well as IL-10 were detected in the BAL cytosol fractions as early as 4 days of age in the group of rats studied. The MW of the cytokines studied coincides with values published by Abbas and Litchtman (2003).

### DISCUSSION

T helper cells can be classified into different types based on their cytokine profile. Cells with these polarized patterns of cytokine production have been termed as Th1 and Th2. Th1 cells produce IL2, INF-γ, TNF-α, lymphotoxin and they promote delayed-type hypersensitivity responses. Th2 cells produce other cytokines such as IL4, IL10, IL5, IL6 and IL13 (Abbas and Litchtman, 2003). The cytokine, most characteristic of Th1 responses is INF-γ, a major component of the cell mediated immune response and inflammation (Openshaw *et al.*, 1995; Nagler-Anderson, 2000). It is a highly versatile homodimeric protein that plays an essential role in cell mediated immune response to viral and mycobacterium infection (Abbas and Litchtman, 2003)

TNF-α is very important in innate immunity and phylogenesis which has been described in human, fish, rabbit, mouse, monkey, cat, sheep, woodchuck, possum, flounder and trout. It is produced as a type II transmembrane protein or glycoprotein, that is cleaved within the extracellular C-terminal domain to release a biologically active mature peptide of 17 kd (Secombes *et al.*, 2001). Three of these polypeptide chains polymerize to form 51 kd circulating TNF-α protein (Abbas and Litchtman, 2003). The active form of TNF-α, homotrimer, binds to two distinct receptors of the cells surface, TNFR1 and TNFR2 (Zou *et al.*, 2003).

IL-10 is a multifunctional cytokine with a principal routine function which appears to be limited and ultimately terminate inflammatory responses. This cytokine plays a key role in differentiation and function of a T regulatory cell which control immune responses and tolerance *in vivo* (Moore *et al.*, 2001).

Cytokine synthesis is generally not constitutive, and only a small proportion of cells has obtained *ex vivo* stain for intracellular cytokines. Intracellular cytokine staining has played an important part in defining functional subsets in mixed T cell populations (Pala *et al.*, 2000). It allows both the frequency of cells producing specific cytokines to be estimated and, to some extent, the levels of expression to be compared. Different cytokines have different kinetics of expression, so that optimal time for detection varies.

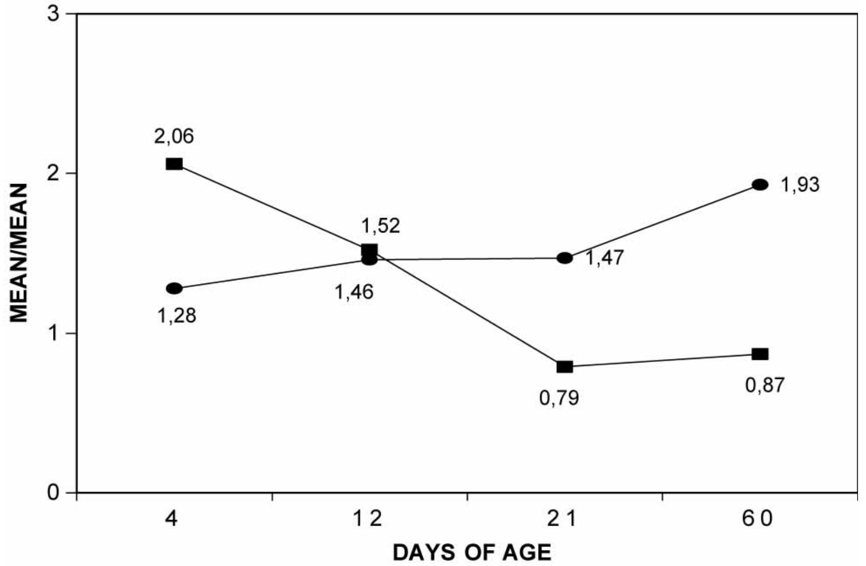

FIGURE 4 Describe the relationship between TNF-a/IL-10 + and IFN g/IL-10 + cells in the BALT of growing rats. In this figure we observe a high TNF-α/IL-10 ratio at day 4, but reverses at day 21. In contrast the IFN-γ/IL-10 ratio is always greater than one.

TABLE II Relationship between TNF-a/IL-10+ cells and IFN-γ/IL-10+ cells in the BALT of growing rats

| | Days of age | | | |
|---|---|---|---|---|
| Ratio | 4 | 12 | 21 | 60 |
| TNF-α/IL-10 | 2.06 | 1.52 | 0.79 | 0.87 |
| INF-γ/IL-10 | 1.28 | 1.45 | 1.47 | 1.93 |

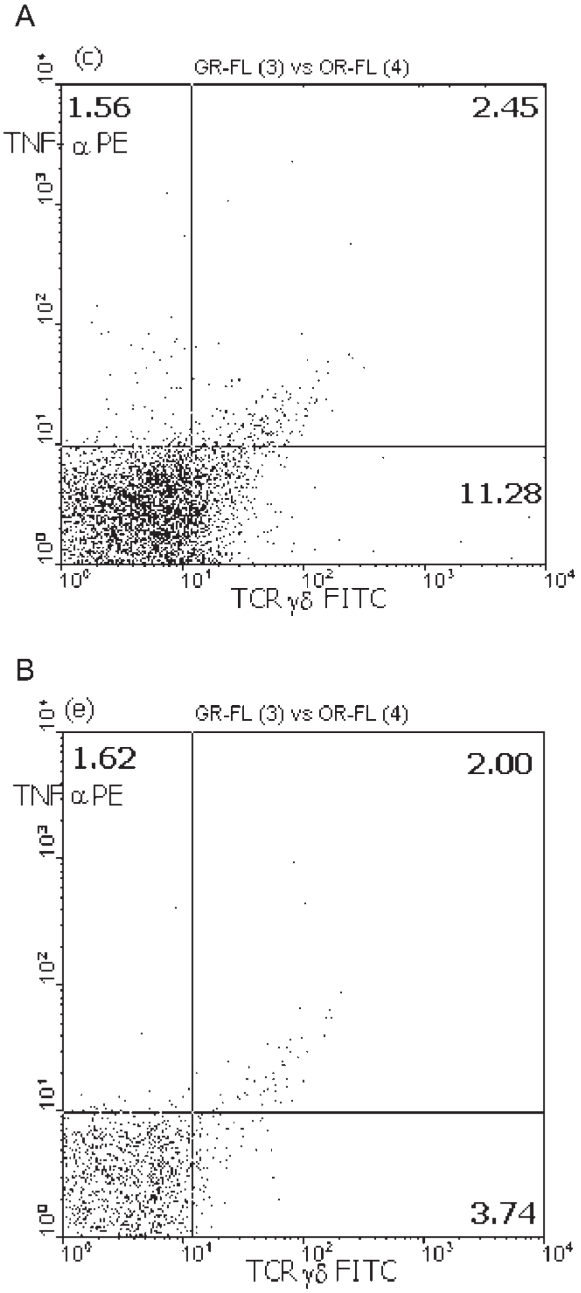

FIGURE 5 Flow cytometry analysis of BAL obtained from 4 and 60 days of age as described in "Materials and Methods" section: (A) 4 days: FITC- TCR γδ TNF-α-PE. (B) 60 days: FITC-TCR γδ TNF-α-PE. Results are represented list 3 replicate experiments. The percentage of cells localized in each quadrant is indicated.

These factors limit the investigation of expression of different cytokines in single cells. However, it is possible to determine how many cells express two or more cytokines at a single point in a time but the sequential expression of cytokines by the same cells is rather difficult. This also applies for cells containing different cytokines that express different T- cell phenotype.

Until now, we have not been able to find any report on BALT rat cytokines. Therefore, in the present report three important cytokines such as TNF-α, INF-γ and IL10, that are produced by Th2 T cells and control inflammation were studied.

All the cytokines studied by different techniques were observed at an early stage of BALT development and their molecular weight were in agreement with what has been described in the literature (Abbas and Litchtman, 2003).

During BALT ontogeny, several cytokines such as TNF-α, INF-γ and IL-10 have been determined in the present study. It is well known that they are able to trigger the development of the determined subsets of T-cells.

The principal cytokine found was TNF-α at 4 days of age in BALT cells although INF-γ and IL-10 are present.

According to our study the three cytokines increase along BALT development. However, when studying the relationship between TNF-α/IL-10 compared to IFN-γ/IL-10, we found a clear difference at the different stages of development. The TNF-α/IL-10 ratio, at 4–21 days of age decrease and is maintained at 60 days of age. In contrast, IFN-γ/IL-10 ratio from 4–21 days of age is always the same as above one and is slightly increased at day 60.

These data suggest an important role of TNF-α in the development of BALT. We may think it is quite important that two cytokines participating in innate immunity appear at the same time in ontogeny, presumably in different quantities as their profile are different along the developmental pathway.

The fact that γδ T cell responses have been noted in many situations in which TNF-α is produced is consistent with the notion that this cytokine is one of the major signals triggering γδ T cell reactivity (Lahn *et al.*, 1998). Moreover, in a previous study in our laboratory, the early appearance—4 days of age—of all T-cell phenotype in BALT especially γδ+ T cell has been described (Marquez *et al.*, 2000).

It is known that γδ T cells proliferate and produce cytokines in many diseases contributing to host resistance against infections and regulating inflammation. The greater susceptibility of γδ T cell for TNF-α stimulation could thus bias T cell responses in favor of γδ T cell reactivity whenever this cytokine is present, or might ensure preferential γδ T cell reactivity at limiting cytokine levels. Since γδ T cells themselves can produce TNF-α, they might also be autostimulatory under appropriate conditions. There is evidence for the role of γδ T cell in up-regulating TNF-α production, suggestive of a feedback loop in which γδ T cells could perpetuate the generation of their own major stimulus.

In the present report, we describe by immunofluorescence in the BALT the colocalization of TNF-α + TCR γδ+ cells as early as 4 and 7 days of age. Moreover, by flow cytometry we have shown the presence of TNF-α positive cells that express the TCR γδ phenotype as early as 4 days of age and also in the BAL from 60 days old rats. Therefore knowing the existence of a mutual regulation between them (Hiromatsu *et al.*, 1992; Fu *et al.*, 1994; Boismenu, 2001), we may propose that both are taking part together in the immunosurveillance at the epithelium level.

### Acknowledgements

The authors thank the Department of Nutrition for the use of animal facilities and Maria Cecilia Mambrin for taking care of the breeding unit. This work was supported by grant PIP 02855 (from CONICET) and B064 from the University of Buenos Aires.

### References

Abbas, A.K. and Litchtman, A.H. (2003) "Section IV Effector Mechanisms of Immune responses, Chapters 11: Cytokines, Chapter 12: Innate Immunity, Chapter 13: Effector Mechanisms of cell-mediated Immunity, Chapter 14: Effector Mechanisms of humoral Immunity, Chapter 15: Immunity to microbes in Cellular and Molecular Immunology", In: Abbas, A.K. and Litchman, A.H., eds, Celluar and Molecular Immunology, 5th ed. (Elsevier Science, USA), pp 241–345.

Biennestock, J., McDermott, M.R. and Clancy, R.L. (1999) "Respiratory tract defenses: role of mucosal lymphoid tissues", 18, In: Ogra, P.L., Mestecky, J., Lamm, M.E., Strober, W., Biennestock, J. and McGhee, J.R., eds, Mucosal Immunology, 2nd ed. (USA Academic Press, San Diego), pp 283–292.

Boismenu, R. (2001) "Natural and pharmacological strategies for the protection of mucosal surfaces", *Mucosal Immunol. Update* **8**, 5–9.

Buckley, A.R., Crowe, P.D. and Haddock, R.D. (1988) "Rapid activation of protein kinasa C in isolated rat liver nuclei by prolactin, a known hepatic mitogen", *Proc. Natl Acad. Sci. USA* **85**, 8649–8653.

Caraher, E.M., Parenteau, M., Gruber, H. and Scott, F.W. (2000) "Flow cytometric analysis of intracellular INF-γ, IL-4 and IL-10 in CD3 + CD4 + T-cells from rat spleen", *J. Immunol. Methods* **244**, 29–40.

Fu, Y., Roark, C.E., Kelly, K., *et al.* (1994) "Immune protection and control of inflammatory tissue necrosis by γδ T cells", *J. Immunol.* **153**, 3101.

Hiromatsu, K., Yoshikai, G., Matsuzaki, S., *et al.* (1992) "A protective role of γ/δ T cells in primary infection with *Listeria monocytogenes* in mice", *J. Exp. Med.* **175**, 49–54.

Laemmli, U.K. (1970) "Cleavage of structural proteins during the assembly of the head of bacteriophage T4", *Nature* **227**, 680–685.

Lahn, M., *et al.* (1998) "Early preferential stimulation of γδ T cells by TNF-α", *J. Immunol.* **160**, 5221–5230.

Marquez, M.G. and Roux, M.E. (1998) "γδ T cell development in the bronchus-associated lymphoid tissue (BALT) of Wistar rats", In: Talwar, G.P., Nath, I., Ganguly, N.K. and Rao, K.V.S., eds, Proceedings of the 10th International Congress of Immunology (Monduzzi Editore S.p.A, Bologna, Italy), pp 1085–1088.

Marquez, G., Sosa, G. and Roux, M.E. (2000) "Developmental study of immunocompetent cells in the bronchus-associated lymphoid tissue (BALT) from Wistar rats", *Dev. Comput. Immunol.* **24**, 683–689.

Moore, K.W., de Waal Malefyt, R., Coffman, R.L. and O'Garra, A. (2001) "Interleukin-10 and the Interleukin-10 receptor", *Annu. Rev. Immunol.* **19**, 683–765.

Nagler-Anderson, C. (2000) "Man the barrier! Strategic defences in the intestinal mucosa", *Nat. Rev. Immunol.* **1**, 59–67.

Nishimura, H.M., Emoto, K., Hiromatsu, S., *et al.* (1995) "The role of γδ T cells in priming macrophages to produce tumor necrosis factor-α", *Eur. J. Immunol.* **25**, 1465–1469.

Openshaw, P., Murphy, E.E., Hosken, N.A., Maino, V., Davis, K., Murphy, K. and O'Garra, A. (1995) "Heterogeneity of intracellular cytokines synthesis at the single-cell level in polarized T Helper 1 and T Helper 2 populations", *J. Exp. Med.* **182**, 1357–1367.

Pala, P., Hussell, T. and Openshaw, P.J.M. (2000) "Flow cytometric measurement of intracellular cytokines", *J. Immunol. Methods* **243**, 107–124.

Sainte-Marie, G.A. (1962) "Paraffin-embedding technique for studies employing immunofluorescence", *J. Histochem. Cytochem.* **10**, 250–256.

Secombes, C.J., Wang, T., Hong, S., Peddie, S., Crampe, M., Laing, K.J., Cunningham, C. and Zou, J. (2001) "Cytokines and innate immunity of fish", *Dev. Comput. Immunol.* **25**, 713–723.

Sminia, T., van der Brugge-Gamelkoorn, G.J. and Jeurissen, S.H.M. (1989) "Structure and function of bronchus-associated lymphoid tissue (BALT)", *Crit. Rev. Immunol.* **9**, 119–150.

Towbin, H., Staehelin, T. and Gordon, J. (1979) "Electrophoretic transfer of proteins from polyacrilamide gels to nitrocellulose sheets: procedure and some applications", *Proc. Natl Acad. Sci. USA* **76**, 4350–4354.

Zou, J., Peddie, S., Scapigliati, G., Bols, N.C., Ellis, A.E. and Secombe, C.J. (2003) "Functional characterization of the recombinant tumor necrosis factor in rainbow trout *Oncorhynchus mykiss*", *Dev. Comput. Immunol.* **27**, 813–822.

